# Comparison of a Powdered, Acidified Liquid, and Non-Acidified Liquid Human Milk Fortifier on Clinical Outcomes in Premature Infants

**DOI:** 10.3390/nu8080451

**Published:** 2016-07-26

**Authors:** Melissa Thoene, Elizabeth Lyden, Kara Weishaar, Elizabeth Elliott, Ruomei Wu, Katelyn White, Hayley Timm, Ann Anderson-Berry

**Affiliations:** 1 Newborn Intensive Care Unit, Nebraska Medicine, 981200 Nebraska Medical Center, Omaha, NE 68198, USA; 2 College of Public Health, University of Nebraska Medical Center, 984375 Nebraska Medical Center, Omaha, NE 68198-4375, USA; elyden@unmc.edu; 3 Department of Pediatrics, University of Nebraska Medical Center, 981205 Nebraska Medical Center, Omaha, NE 68198-1205, USA; kara.weishaar@unmc.edu (K.W.); elizabeth.elliott@unmc.edu (E.E.); Ruomei.Wu@Creighton.edu (R.W.); katelyn.white@huskers.unl.edu (K.W.); hayley.timm@grmep.com (H.T.); alanders@unmc.edu (A.A.-B.)

**Keywords:** human milk, fortifier, premature infant, enteral nutrition, growth, acidosis, necrotizing enterocolitis

## Abstract

We previously compared infant outcomes between a powdered human milk fortifier (P-HMF) vs. acidified liquid HMF (AL-HMF). A non-acidified liquid HMF (NAL-HMF) is now commercially available. The purpose of this study is to compare growth and outcomes of premature infants receiving P-HMF, AL-HMF or NAL-HMF. An Institutional Review Board (IRB) approved retrospective chart review compared infant outcomes (born < 2000 g) who received one of three HMF. Growth, enteral nutrition, laboratory and demographic data were compared. 120 infants were included (P-HMF = 46, AL-HMF = 23, NAL-HMF = 51). AL-HMF infants grew slower in g/day (median 23.66 vs. P-HMF 31.27, NAL-HMF 31.74 (*p* < 0.05)) and in g/kg/day, median 10.59 vs. 15.37, 14.03 (*p* < 0.0001). AL-HMF vs. NAL-HMF infants were smaller at 36 weeks gestational age (median 2046 vs. 2404 g, *p* < 0.05). However AL-HMF infants received more daily calories (*p* = 0.21) and protein (*p* < 0.0001), mean 129 cal/kg, 4.2 g protein/kg vs. P-HMF 117 cal/kg, 3.7 g protein/kg , NAL-HMF 120 cal/kg, 4.0 g protein/kg. AL-HMF infants exhibited lower carbon dioxide levels after day of life 14 and 30 (*p* < 0.0001, *p* = 0.0038). Three AL-HMF infants (13%) developed necrotizing enterocolitis (NEC) vs. no infants in the remaining groups (*p* = 0.0056). A NAL-HMF is the most optimal choice for premature human milk-fed infants in a high acuity neonatal intensive care unit (NICU).

## 1. Introduction

Premature infants have significantly increased nutrient needs compared to those born at term [1]. Nutrition-related goals for these infants must aim at promoting similar nutrient provision and growth as that achieved in utero. Providing human milk remains a preferable nutrient source over customized premature infant formulas, but alone remains inadequate to meet the high nutritional needs for rapid growth and development. Long term provision of unfortified human milk has been linked to suboptimal growth, poor bone mineralization, and multiple nutrient deficiencies of vitamins, minerals, and trace elements [1]. As a result, human milk fortifiers (HMF) are used to significantly enhance calorie, protein, vitamin, and mineral intake of the human milk fed premature infant.

Enteral macronutrient recommendations for premature infants vary according to size. The American Academy of Pediatrics on nutrition suggests 130–150 calories/kilogram (kg) and 3.8–4.4 g protein/kg/day for infants weighing <1000 g, and 110–130 calories/kg and 3.4–4.2 g protein/kg/day for infants weighing between 1000 and 1500 g [2]. Protein is highly emphasized as high adequate provision has been correlated with improved growth and neurodevelopment [3,4,5,6]. To achieve enteral protein goals, powdered or liquid protein modulars may be added alongside HMF to optimize overall nutrition.

Human milk fortifiers are available in many different compositions, specifically varying in protein type, protein amount, and form (powder vs. liquid). However if available, the Food and Drug Administration strongly recommends the use of liquid products over powder in the neonatal intensive care unit (NICU) setting in an effort to reduce contamination and infection risk [7]. Our unit originally used a powdered HMF, but transitioned to liquid form when they became commercially available. Previously, we published a study comparing two HMF used in our unit, one being a powder (P-HMF) and one being an acidified liquid (AL-HMF) [8]. Our results demonstrated that the acidified product, though sterile, caused more metabolic acidosis and poor growth in our population of premature infants. Infants receiving the AL-HMF also had a higher incidence of necrotizing enterocolitis (NEC), though we were not powered to find this. Our unit has now transitioned to using a non-acidified liquid fortifier (NAL-HMF). The purpose of this study is compare growth and clinical outcomes of infants receiving this new HMF to the previous two fortifier groups. 

## 2. Patients and Methods

### 2.1. Participants and Data Collection

The institutional review board at the University of Nebraska Medical Center (Omaha, NE, USA) approved this study. Data was retrospectively collected from inpatient electronic medical records of all infants admitted to the NICU between August 2012 and July 2014 if they met the following criteria; birth weight (BW) < 2000 g, received at least 25% of enteral feedings as fortified human milk (with the NAL-HMF) during their NICU stay, and remained in the NICU at least 14 days. Exclusion criteria included infants with congenital abnormalities or conditions that inhibited growth, such as Trisomy 13. No infants were excluded based on clinical acuity, intrauterine growth restriction, APGAR score, or ventilator requirements.

Data on the P-HMF and AL-HMF groups was previously collected for infants admitted to the NICU between October 2009 and July 2011. The AL-HMF group contained a lower number of included infants due to this HMF being used for a limited time period. Six investigators familiar with the electronic medical record obtained all data for the NAL-HMF group in a similar manner as the original groups. Data was reviewed closely for accuracy and corrected if an electronic error occurred. Available data on each infant was included in the analysis and is displayed in the tables.

### 2.2. Demographics and Clinical Outcomes

Demographic information was collected for all infants including gender, gestational age at birth and discharge, and day of life (DOL) at discharge. Additional clinical outcomes were collected as available including presence of bronchopulmonary dysplasia (BPD) defined as oxygen requirement at 36 weeks estimated gestational age (EGA), retinopathy of prematurity (ROP) Stage 2 or greater, Grade 3 or 4 intraventricular hemorrhage (IVH), NEC, and death. Treatment requirements were also analyzed including need for intraventricular shunt, ROP procedure, and Dexamethasone use.

### 2.3. Growth and Nutrition

Infants were weighed daily on a gram (g) scale, and length and head circumference measurements (cm = centimeters) were taken weekly using a measuring tape by nursing staff. Percentile rankings from the Fenton growth chart were electronically plotted for each documented measurement. Weight, length, and head circumference measurements were recorded for infants at birth and 36 weeks EGA if still hospitalized. An EGA of 36 weeks was empirically selected as an equivalent point of analysis for growth prior to discharge.

Enteral feeding data collected included DOL enteral feedings were initiated and DOL full enteral feedings were reached. Full enteral feedings was defined as the infant receiving at least 140 milliliters (mL)/kilogram (kg)/day of fortified enteral feedings and no parenteral nutrition. Average calorie and protein intake measured in per kg/day was analyzed for infants who received at least 50% of their feedings as fortified human milk during NICU stay. Intake was analyzed from the start of full feedings until the HMF was discontinued or the infant received <50% of feedings as fortified milk. Growth as measured in g/day and g/kg/day was calculated for infants during the time of reaching full enteral feedings until they received <50% of feedings as fortified milk. Maximum caloric density of feedings was recorded for each infant. Number of days on caloric densities higher than the standard 24 calories/ounce was collected for infants requiring more to maintain growth chart percentiles for weight. Nutrient provision was captured by an electronic medical system (Intuacare), which contained protein references based on the caloric density of specified formulas or fortified human milk. Nursing staff recorded daily intake (in mL) of specified feedings, and daily calorie and protein per kilogram was electronically calculated using the daily recorded weight. The electronic system also calculated the percentage of human milk vs. infant formula received according to nursing documentation.

### 2.4. Comparison and Use of Human Milk Fortifiers

Comparison of ingredients and key HMF nutrients are listed in Table 1 according to online nutritional references [9,10,11].

Enteral feeding are initiated in this NICU as soon as able following birth, within the first one to three days of life using maternal breast milk (MBM) as available or donor human milk (from the Milk Bank of Austin, Texas) at 20 mL/kg/day. Trophic feedings are continued for three to five days at the discretion of the attending neonatologist. Feedings are then advanced by 20 mL/kg/day and HMF is added when enteral volumes feedings reach 80–100 mL/kg. A protein modular is also added once caloric densities reach 24 calories/ounce to optimize protein intake to approximately 4 g/kg/day. The calories provided from the protein modular are accounted for in the calorie-per-ounce estimates. The P-HMF group received a powdered protein modular and the NAL-HMF group received a liquid protein modular. No additional protein modular was provided to infants receiving the AL-HMF due to higher protein content of the fortifier. All infants are transitioned off of donor human milk to 24 calorie/ounce high protein (3.5 g protein per 100 calories) premature infant formula at 14 days of life if a supplement to MBM is needed. We did not analyze differences in donor human milk use between groups because it is only used for a short period after birth and is provided to all infants in a similar manner. There were no other nutrition practice changes during the periods of different fortifier use. Our unit follows a written feeding protocol, so nutrition is managed closely and remains consistent among providers.

### 2.5. Laboratory Measurements

Lowest carbon dioxide (CO_2_) lab values were collected after DOL 14 and 30 for all infants, if available. Values were not collected prior to eliminate values reflective of parenteral nutrition support and unfortified enteral feedings. Maximum blood urea nitrogen (BUN) while on full enteral feedings was additionally collected.

### 2.6. Data Analysis

The Kruskal Wallis test was used to compare continuous data between the three HMF groups. If the overall p-value was significant, indicating a significant difference between at least two of the three groups, the Dunn’s post hoc test for three pair wise comparisons (i.e., Group 1 vs. 2, Group 1 vs. 3, Group 2 vs. 3) was performed. Associations of categorical variables were assessed with the Fisher’s exact test. Time to weaning off oxygen distributions were estimated using the method of Kaplan and Meier and were compared using the log-rank test. A *p*-value < 0.05 was considered statistically significant.

To assess the difference in growth patterns between infants, a mixed effects model was used. We included random slopes and intercepts for each subject to capture individual growth pattern as well as fixed effects for group and day and a group day interaction term. A significant interacting of day and group indicated differing growth patterns based on group. Growth Velocity (GV) was calculated using the following equation [12]:

GV = [1000 × ln(Wn/W1)]/(D*_n_* − D_1_) (1)

Where W*_n_* refers to the weight on the last evaluated day; W_1_ refers to the first weight; D*_n_* refers to the last day of the time period evaluated and; D_1_ refers to the first day of the time period evaluated.

## 3. Results

There were 46 infants in the P-HMF, 23 in the AL-HMF, and 51 in the NAL-HMF groups. There were no significant differences in gender (*p* = 0.6) or baseline characteristics as shown in Table 2. Clinical outcomes are displayed in Table 3. Laboratory, growth, and nutrition data are displayed in Table 4.

### 3.1. Clinical Outcomes

All laboratory data analyzed for this study was collected for clinical purposes. Median lowest C02 levels while on full enteral feedings were significantly lower in the AL-HMF group compared to the other two groups after both DOL 14 and DOL 30 (*p* < 0.0001, *p* = 0.0038). Maximum BUN levels on full enteral feedings were similar among all groups and were not statistically significant. 

The incidence of NEC was significantly higher in the AL-HMF group compared to the P-HMF and NAL-HMF groups (13% vs. 0% and 0%, *p* = 0.0056), though we were not powered to evaluate this variable. Incidence of ROP was significantly higher among the P-HMF than the NAL-HMF group (35% vs. 8%, *p* = 0.003). There were no differences in rates of BPD or IVH (Grade 3 or 4) among all groups.

### 3.2. Enteral Growth and Nutrition

Growth, as measured in both g/day and g/kg/day, was statistically significant between groups. More specifically, infants in the AL-HMF group grew slower than infants in the P-HMF and NAL-HMF groups. Median growth in g/day from start of full enteral feedings until 36 weeks EGA was 23.66, compared to 31.27 in the P-HMF and 31.74 in the NA-LHMF group (*p* = 0.0001). Median growth in g/kg/day was 10.59 in the AL-HMF group, compared to 15.37 and 14.03 respectively (*p* < 0.0001). Infants in the AL-HMF group were smaller at 36 weeks EGA compared to the NAL-HMF group (median 2046 g vs. 2404 g, *p* = 0.0092), though there were no differences in length or head circumference. There were no differences in Dexamethasone use among groups (*p* = 0.15) that may account for reduced growth. Infants in the NAL-HMF group started enteral feedings and achieved full enteral feedings faster than the P-HMF group (*p* = 0.0019, *p* = 0.0007), but these infants achieved similar growth. 

Among infants receiving >50% of their feedings during NICU stay as fortified human milk , infants in the AL-HMF group received more protein at mean 4.2 g/kg/day compared to 3.7 and 4.0 g/kg/day in the P-HMF and NAL-HMF groups (*p* < 0.0001). These infants also received a higher mean calorie intake at 129 calories/kg, compared to 117 and 120 calories/kg, respectively, though this was not significant (*p* = 0.21).

There were no differences in maximum caloric density of enteral feedings (*p* = 0.6) or the number of days on feedings >24 calories/ounce (*p* = 0.21). Noted however, is that 48% of infants in the AL-HMF group received enteral feedings >24 calorie/ounce compared to 26% in the P-HMF group and 35% in the NAL-HMF group.

## 4. Discussion

Our previous research analyzing the P-HMF and AL-HMF suggested the P-HMF was the more optimal choice in promoting best clinical outcomes [8]. Now comparing data among all three fortifier groups, the NAL-HMF appears to be the most successful fortifier for use in a high acuity NICU population. Despite achieving adequate similar growth, the NAL-HMF is more desirable than the P-HMF due to its composition as a sterile liquid. When compared to the AL-HMF, the NAL-HMF promoted greater growth and was not associated with metabolic acidosis or NEC. 

### 4.1. Growth and Enteral Nutrition

Appropriate growth was best achieved among the NAL-HMF and P-HMF groups when comparing both g/day and g/kg/day weight gain. Infants receiving the NAL-HMF attained the highest weight among all three groups at 36 weeks EGA, demonstrating most significance when compared to the AL-HMF group (median 2046 g vs. 2404 g, *p* < 0.0092). In further comparison of this, the median length and head circumference for both the acidified and non-acidified liquid group at this point plotted between the 25%–30% on the Fenton growth chart. By comparison of median weights at 36 weeks, the NAL-HMF group plotted around the 18% and the AL-HMF group plotted at the 5th. This demonstrates that infants receiving the NAL-HMF were able to achieve a more proportional weight-for-length ratio. Though we did not directly assess infant acuity level between groups, we do not suspect this to be a significant factor for decreased growth given the AL-HMF group having similar baseline characteristics as the other groups.

As growth remains a high priority, infants with suboptimal growth were fed enteral feedings with caloric densities >24 calories/ounce. Suboptimal growth was determined by clinical evaluation when an infant was unable to maintain growth percentiles for weight. Despite decreased growth, more infants in the AL-HMF (48%) group required increased caloric density of feedings compared to the P-HMF (26%) and NAL-HMF (35%) groups, *p* = 0.6. The AL-HMF group also received higher mean calorie intake compared to the other two groups. Had no infants been advanced to increased caloric densities, it is likely that the discrepancy of growth between the AL-HMF and the remaining two groups would have been of even greater significance. It may be theorized that additional enteral additives and higher caloric densities contributed to a higher incidence of NEC in the AL-HMF group. However, our previously low recorded rate of NEC at 3% is reflective of similar fortification practices to achieve desired growth [13]. Despite individual theories for these NEC occurrences, we must address why the additional additives were required in the first place to achieve adequate growth.

In additional to increased calories, infants in the AL-HMF group also received a higher mean protein intake compared to the other groups. Higher protein provisions have been linked to improved growth, yet these infants exhibited poor weight gain. We hypothesize that the acidification of the AL-HMF may be the explanatory factor in this conundrum. A study by Erickson et al. concluded that acidifying human milk resulted in 14% decrease in protein and a 56% decrease in lipase activity [14]. This may result in partial fat malabsorption and resulting poor energy intake. A recent study by Cibulskis and Armbrecht comparing infants receiving an acidified vs. powdered HMF did not report significant growth differences in weight, length, or head circumference between birth and discharge [15]. However, growth measured in g/day while on the HMF approached significance as infants receiving the acidified HMF grew slower (22.3 vs. 19.2 g/day, *p* = 0.08). In comparison, Moya et al. reported no discrepancies in weight gain when comparing infants ≤1250 g receiving either an acidified or powdered HMF, and further reported that infants receiving the acidified HMF had improved linear growth [16]. Limitations of this study, however, include that protein modulars were used infrequently among infants, so baseline protein provisions were higher in the acidified HMF group. This study also excluded infants with low APGAR scores and higher respiratory requirements so may not be applicable to the most fragile infants.

### 4.2. NEC

The only infants who developed NEC received the AL-HMF. Though not statistically powered to find NEC, the results raise concern from a clinical standpoint. Our feeding practices have remained consistent outside of which HMF was used, and we have documented low baseline rates of NEC on these feeding practices [13]. Feeding initiation and advancement remained fairly consistent across all three groups. While infants in the NAL-HMF group achieved full feedings more quickly, none developed NEC. Formula was utilized equally in all groups when MBM was limited and donor human milk was weaned. 

The primary differences in enteral feedings between all fortifier groups are the acidity, high protein, and high iron content of the AL-HMF. Theoretically, infants receiving the AL-HMF had a reduced risk for cross-contamination due to the HMF composition as a sterile liquid and because additional enteral substrates (protein modular, iron) were not required. These infants also received lower osmolality feedings at baseline, and furthermore as additional supplements were not required due to the high iron and protein in the AL-HMF. A study by *Chan* suggests that a high iron-containing HMF compared to a low iron-containing HMF negates the antimicrobial effects of human milk against the growth of *E. coli*, Staphylococcus, Enterobacter, and Streptococcus [17]. Erickson et al. also noted a reduced white cell count by 76% in human milk acidified to a pH of 4.5, questioning if this decreases an infant’s host defense [14]. The AL-HMF used in our study acidifies milk similarly to a pH of 4.7. We must consider if the protective effects of human milk were compromised in infants receiving the AL-HMF, making them more susceptible to infections. A limitation to this theory is that we did not analyze the incidence of sepsis between groups. As the cause of these NEC occurrences remains unknown, we can neither confirm nor exclude use of the AL-HMF as a primary contributor.

### 4.3. Acidosis

There was a higher incidence of metabolic acidosis in the AL-HMF group compared to the other two groups. As discussed in our previous study, premature infants are at risk for developing metabolic acidosis secondary to immature renal and metabolic processes [8]. There were no significant differences in baseline characteristics such as birth weight or gestational age to suggest any of the three groups included infants that were smaller or born more prematurely, and therefore more obviously susceptible to acidosis. We do not suspect protein provision as a contributor to acidosis. While infants receiving the AL-HMF received higher daily protein (*p* < 0.001), mean values remained within the reference ranges for very low birth weight infants of 3.4–4.4 g protein/kg/day [2]. BUN levels also remained similar among groups.

While the P-HMF and NAL-HMF do not have defined pH values as shown in Table 1, we suspect they have limited effects on the final pH of fortified milk, unlike the AL-HMF. Considering a similar baseline of other characteristics, we again hypothesize that the acidification of the AL-HMF contributed to this metabolic imbalance. Our results are concurrent with Cibulskis and Armbrecht who reported a higher incidence of metabolic acidosis (54% vs. 10%) in infants <32 weeks EGA or <1500 g receiving an acidified vs. powdered HMF [15]. Moya et al. also reported a lower pH at day of life 14 (*p* = 0.004) and lower carbon dioxide levels at both day of life 14 (*p* < 0.001) and 30 (*p* = 0.021) in infants ≤1250 g receiving an acidified HMF [16]. 

Development of metabolic acidosis may also contribute to altered weight gain and poor nutritional consequences. A small study by Rochow et al. reported lower weight gain (median 9 vs. 21 g/kg/day, *p* < 0.01) in infants <34 weeks EGA who developed metabolic acidosis compared to those who remained unaffected [18]. It was also reported that infants who developed metabolic acidosis had a lower bone density at discharge. Likewise, an early study by Kalhoff et al. analyzed urinary excretion of minerals in premature infants, concluding that a higher amount of calcium and phosphorus is excreted during metabolic acidosis [19]. Resultantly, we suggest using a NAL-HMF to provide appropriate growth, without increasing risk for metabolic acidosis and suboptimal nutrient accretion.

### 4.4. Strengths and Limitations

This study is the first to quantify nutrition and growth outcomes of three HMF in a Level IIIc NICU. We did not exclude infants based on acuity, such as presence of IVH, need for high ventilatory settings, or low APGAR scores. Our high inclusion is more reflective of a standard NICU population, and therefore provides genuine outcomes for both high and moderate acuity infants. This is both relevant and applicable to current NICU settings. Nutrition is managed closely and consistently in our unit, and our current nutrition practices have been published demonstrating excellent growth and low baseline rates of NEC [13]. Additionally unique to our study is the use of protein modulars to provide infants similar protein provisions at baseline (approximately 4 g protein/kg/day when receiving 120 calories/kg/day), and reducing this as a significant confounding factor across fortifier groups.

Limitations of this study include that it is retrospective, and there is a limited number of subjects in the AL-HMF group due to its short term use. Additionally included is our reliance on electronic documentation for data collection, as we cannot quantify unrecorded or misrecorded data. However, the system does allow for review of daily entered data for each subject if needed. Evaluation of head circumference and length measurements may vary among nursing staff due to differences in measuring tape placement. Additionally, growth measurements were unavailable for infants discharged prior to 36 weeks EGA. Growth at 36 weeks EGA may also be partially reflective of formula use if MBM was no longer available. However, it may also provide indication of early growth failure while on MBM if growth percentiles are low or fall drastically from those at birth. The calculated provision for calories and protein in fortified human milk were estimated according to manufacturer information for each HMF. These may only serve as general estimates for our comparisons as the composition of human milk varies continuously. While standard NICU practices remain consistent, feedings may be advanced differently based on each infant’s clinical status. Length of trophic feedings may also impact the day of life to achieving full enteral feedings. As in our previous study of the original two fortifiers, NEC was statistically significant despite our limited power to find this. 

## 5. Conclusions

The NAL-HMF is an appropriate choice for use in a high level NICU. Caution should be taken when using an acidified HMF due to its potential effects on growth, tolerance, and metabolic acidosis.

## Figures and Tables

**Table 1:** Comparison of primary nutrients and ingredients of the powdered, acidified liquid, and non-acidified liquid HMF.

24-Calorie-Per-Ounce Fortified Human Milk [9,10,11]			
Per 100 mL	P-HMF	AL-HMF	NAL-HMF
Protein (g)	2.35 g	3.2 g	2.34 g
Iron (mg)	0.46 mg	1.85 mg	0.46
Calcium (mg)	138 mg	141 mg	138
Phosphorus (mg)	78 mg	78 mg	77
Vitamin D (IU)	119 IU	200 IU	118
pH	---	4.7	---
Osmolality (mOsm/kg water)	385	326	385
Primary Fortifier Macronutrient Ingredients	nonfat milk, whey protein concentrate, corn syrup solids, medium-chain triglycerides (MCT oil)	water, whey protein isolate hydrolysate (milk), medium chain triglycerides (MCT oil), vegetable oil (soy and high oleic sunflower oils)	water, nonfat milk, corn syrup solids, medium-chain triglycerides (MCT oil), whey protein concentrate

-- Information not available. P-HMF, powdered human milk fortifier; AL-HMF, acidified liquid HMF; NAL-HMF, non-acidified liquid HMF.

**Table 2:** Baseline characteristics of subjects by group.

Variable	P-HMF (Group 1)		AL-HMF (Group 2)		NAL-HMF (Group 3)		Overall *p*-Value
	*n*	Median	*n*	Median	*n*	Median	
EGA at Birth	46	29.15	22	31.00	51	29.60	0.15
Birth Weight (g)	46	1305	22	1481	51	1340	0.21
Weight at 36 Weeks EGA (g)	44	2179	18	2046	50	2404	0.0092 Group 2 vs. 3 *p* < 0.05
Birth Length (cm)	46	39	22	41	51	39	0.14
Length at 36 Weeks EGA (cm)	42	44.5	18	43.5	47	44	0.38
Birth HC (cm)	46	27	22	27.75	51	27.5	0.53
HC at 36 Weeks EGA (cm)	42	32.5	18	31.75	47	32.2	0.55

EGA = Estimated Gestational Age; HC = Head Circumference.

**Table 3:** Clinical outcomes of subjects by group.

Variable	P-HMF (Group 1) *n* = 46	AL-HMF (Group 2) *n* = 23	NAL-HMF (Group 3) *n* = 51	Overall *p*-Value
	*n (%)*	*n (%)*	*n (%)*	
NEC	0	3 (13%)	0	0.0056
ROP	16 (35%)	3 (13%)	4 (8%)	0.0030 Group 1 vs. 3, *p* = 0.006
ROP Procedure	3 (7%)	2 (9%)	1 (2%)	0.24
IVH (Grade 3 or 4)	3 (7%)	1 (5%)	4 (8%)	1.00
Intraventricular Shunt	0	0	0	N/A
Dexamethasone Treatment	9 (20%)	1 (5%)	7 (14%)	0.29
Death	0	0	1 (2%)	1.00
BPD	10/40 (25%)	4/18 (22%)	16/49 (33%)	0.65

**Table 4:** Laboratory, growth, and enteral nutrition data.

Variable	P-HMF (Group 1)		AL-HMF (Group 2)		NAL-HMF (Group 3)		Overall *p*-Value
	*n*	Median	*n*	Median	*n*	Median	
Mean Daily Calorie Provision (per kg)	42	117	18	129	48	120	0.21
Mean Daily Protein Provision (g/kg)	42	3.7	18	4.2	48	4.0	0.0001 Group 1 vs. 2 and Group 2 vs. 3, *p* <0.05
Day of Life Feedings Started	46	1	22	1	51	1	0.0019 Group 1 vs. 3 *p* < 0.05
Day of Life Full Feedings Achieved	46	12	22	10	51	9	0.0007 Group 1vs. 3 *p* < 0.05
Growth on HMF (g/day)	45	31.27	21	23.66	49	31.74	0.0001 Group 1 vs. 2 and Group 2 vs. 3, *p* < 0.05
Growth on HMF (g/kg/day)	45	15.37	21	10.59	49	14.03	<0.0001 Group 1 vs. 2 and Group 2 vs. 3, *p* < 0.05
BUN Maximum on Full Feedings	33	17	17	19	47	16	0.43
CO_2_ Minimum after DOL 14	33	23	17	19	32	27	<0.0001 Group 1 vs. 3 and Group 2 vs. 3, 0.05
CO_2_ Minimum after DOL 30	23	25	9	20	18	25.5	0.0038 Group 1 vs. 2 and Group 2 vs. 3, *p* < 0.05
